# Improving equity in human genomics research

**DOI:** 10.1038/s42003-022-03236-9

**Published:** 2022-03-24

## Abstract

Despite recent advances in genotyping technologies, cohorts outside of European ancestries are still underrepresented in genome-wide association studies (GWASs). We recognize the scientific imperative of improving diversity in human genomics, and outline the steps we will take as a journal to empower research involving these underrepresented cohorts.

Genome-wide association studies (GWASs) are an important tool in probing the genetic basis of complex traits and identifying potential targets for disease therapeutics. While the resolution and cost-efficiency of genotyping technologies have steadily improved over time, making it possible to survey more individuals than ever before, the majority of published GWASs still prioritize populations with European ancestry^1,2^. As of January 2022, the GWAS Diversity Monitor estimates that 95.82% of all studies curated through the NHGRI-EBI GWAS Catalog involve cohorts with European ancestry, followed by 3.05% Asian ancestry, and <1% each for African, African American/Afro-Caribbean, Hispanic/Latin American, or other ancestries^3^. This overarching lack of diversity in genomics research is detrimental to our understanding of human biology, especially when considering that multi-ethnic cohorts can help discover rare variants associated with complex traits^1^. Humanity is also not a monolith, and certain genetic variants are ancestry-specific, raising the question of whether existing genomic resources accurately reflect the health needs for diverse populations^1–3^.

It is our responsibility as a journal to establish clear policies that will help advance genomics research, especially studies that focus on these underrepresented populations.

As we highlighted in our 2021 DNA Day Collection, *Communications Biology* is proud to publish studies that showcase the importance of genomics research in underrepresented or multi-ethnic cohorts. However, we recognize that authors working with underrepresented cohorts still face many barriers to publication. It is our responsibility as a journal to establish clear policies that will help advance genomics research, especially studies that focus on these underrepresented populations. After careful discussion with an advisory board of expert human genomics researchers, we have developed the following guidelines:

1. We will not assess studies just based on their cohort size. It is unfair to enforce an arbitrary cutoff or range of sample sizes to any GWAS, whole-genome, or whole-exome sequencing study, especially when considering common vs. rare disorders. Instead, we will consider whether the study itself has sufficient statistical power to identify variants at genome-wide significance.
2. There is biological value in confirming whether significant variants are shared between ancestries. Since launching in January 2018, part of our mission has been to publish “basic research that provides the raw material for future technological and medical breakthroughs”^4^. To that end, we believe that it is an important advance to confirm whether variants discovered in cohorts with European ancestry are also enriched in cohorts with distinct ancestries. Alternatively, we could consider an appropriately-powered GWAS that fails to replicate a variant associated with European ancestry using an underrepresented cohort. Although on a case-by-case basis, we may also consider appropriately-powered GWASs with null results, as these studies may pave the foundation for future meta-analyses.
3. We will be flexible in terms of data availability. Our data availability policy typically requires that all raw sequencing data and GWAS summary statistics be made immediately available upon publication. However, we recognize that this policy may be disadvantageous to researchers in developing countries, who often lack the computational resources to quickly analyze datasets. We are willing to consider exceptions to this immediate data availability policy in cases where it is clear that future projects or publications from local researchers will suffer without a period of exclusive access to the underlying datasets.
4. Diversity behind the bench is also important. In addition to developing diverse genomic resources, it is important for researchers to help train the next generation of scientific leaders. We strongly encourage authors to engage with local researchers and provide trainees with hands-on experience in data collection, analysis, and writing manuscripts or funding proposals. These collaborations should be outlined in the Author Contributions statement, or provided as Diversity, Equity, and Inclusion statements, similar to the Citation Diversity statements present in some of our publications^5,6^. At the same time, we ask that authors establish clear plans to share their findings with local communities post-publication^7^, and are happy to discuss other ways that our journal platform can disseminate any findings.
5. We will continue to direct authors to resources for publishing open-access. The cost of open access publishing can be prohibitive for many authors in developing countries, and Springer Nature offers publishing fee waivers or discounts when corresponding authors are based in the world’s lowest income countries, as defined by the World Bank. As editors, we are not involved in the financial process, though we encourage authors to explore our Open Research Funding & Support Services page, which provides additional resources to apply for waivers to article processing charges.

It is important to note that these criteria are not meant to be a free pass, but to set expectations of what we would consider in any human genomics study. Any work that is submitted to *Communications Biology* must still provide appropriately-powered, well-controlled data that accounts for common experimental confounds. However, we hope that these points will provide clearer guidance to prospective authors, and streamline the path to publication for works involving underrepresented cohorts. We remain excited to provide a home for human genomics research, while improving equity and diversity in genomics resources.

## Advisory board

We are truly grateful to the researchers who participated in discussions to establish these guidelines, and have listed them below in alphabetical order by surname. Members of the *Communications Biology* editorial board are denoted with asterisks (*).

Elizabeth Atkinson, Hélène Choquet*, Chiea Chuen Khor*, and Ambroise Wonkam.
